# Supplementary figures and images for: Transposon mutagenesis reveals Pseudomonas cannabina pv. alisalensis optimizes its virulence factors for pathogenicity on different hosts
Source: PeerJ. 2019 Sep 20;7:e7698. doi: 10.7717/peerj.7698 (PMC6756136; doi:10.7717/peerj.7698)

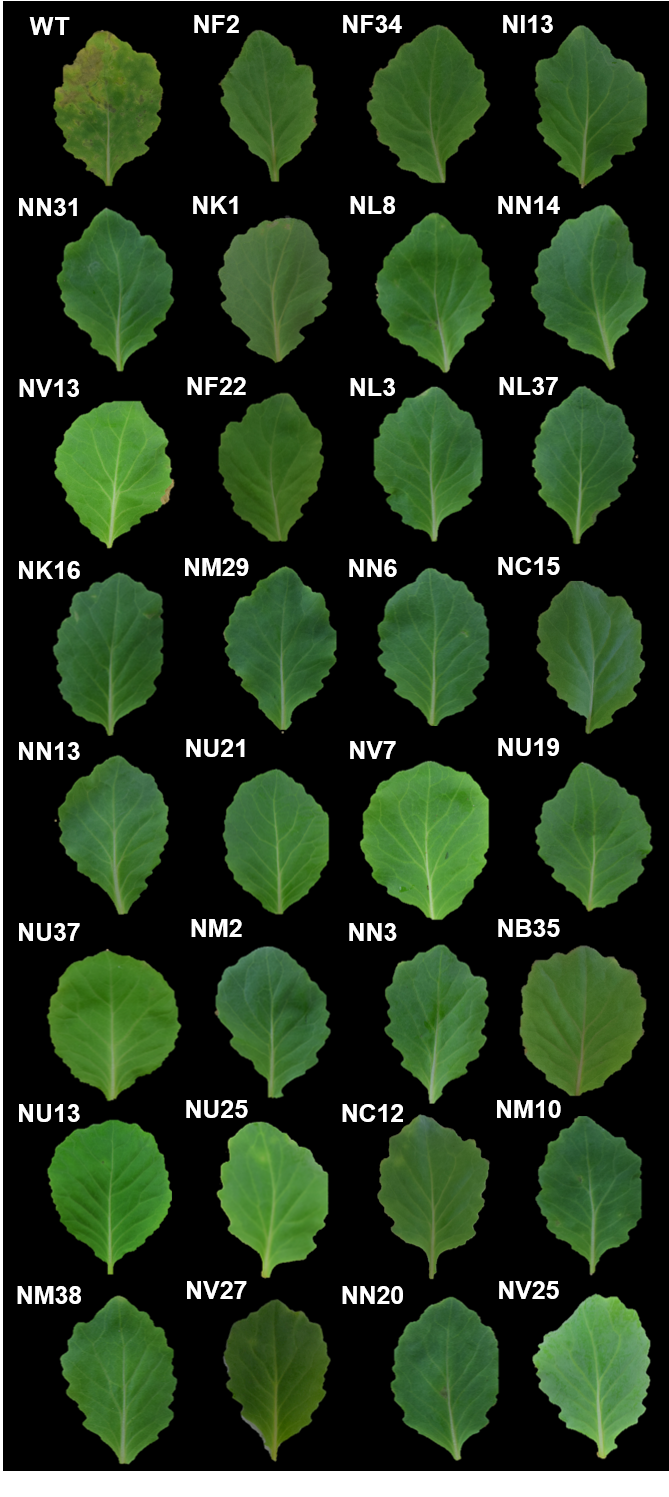

Supplement: Figure S1 — Seedlings of cabbage were inoculated with wild-type Pcal and mutants containing 0.025% Silwet L-77 at a concentration of 5 ×10^7^ CFU/ml. Photograph was taken 5 days post-inoculation. [file peerj-07-7698-s001.png]

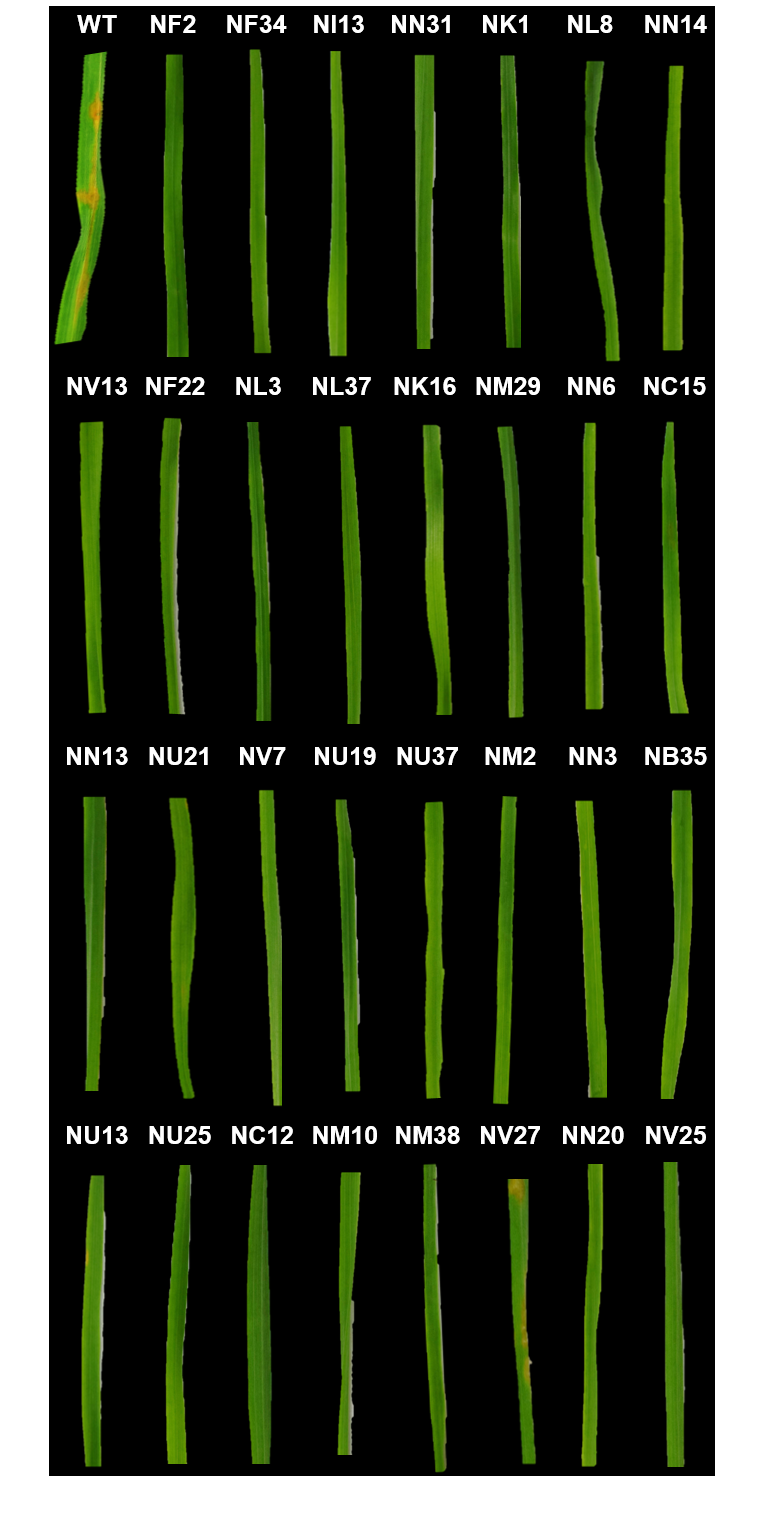

Supplement: Figure S2 — Seedlings of oat were inoculated with wild-type Pcal and mutants containing 0.025% Silwet L-77 at a concentration of 5 ×10^7^ CFU/ml. Photograph was taken 4 days post-inoculation. [file peerj-07-7698-s002.png]

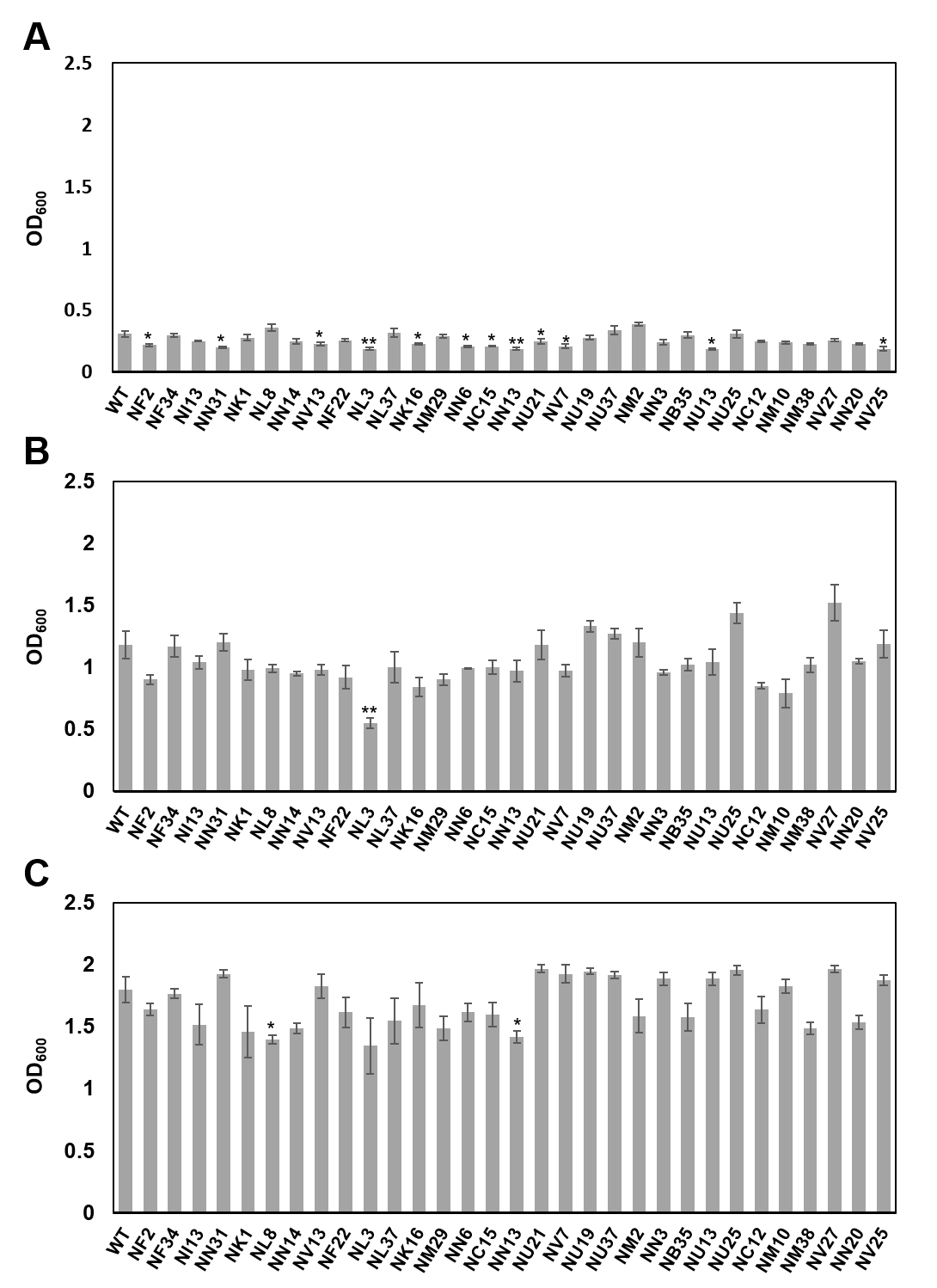

Supplement: Figure S3 — Cultures were adjusted to OD600 = 0.01 and incubated with shaking at 28. Bacterial populations were quantified at 12 h, 24 h, and 48 h. Vertical bars indicate the standard error for three independent experiments. Asterisks indicate a significant difference from the WT and each mutant in a t test (* <0.05, ** <0.01). [file peerj-07-7698-s003.png]

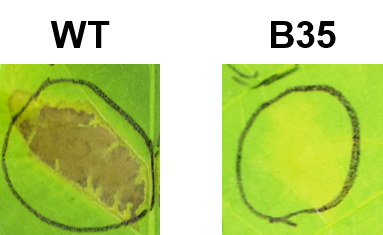

Supplement: Figure S4 — Bacterial suspensions (5 ×10^7^ CFU/ml) of Pcal wild-type and T3SS mutant (B35) were infiltrated into leaves using a 1 ml needleless syringe. HR cell death was observed 24 hours after infiltration. [file peerj-07-7698-s004.png]
